# Taxonomic Status and First Record of a Yellow-Flowered *Astragalus* (sect. *Dissitiflori*, Fabaceae) from Central Balkans

**DOI:** 10.3390/plants15182807

**Published:** 2026-09-13

**Authors:** Predrag Lazarević, Eva Kabaš, Snežana Vukojičić, Maja Lazarević

**Affiliations:** Institute of Botany and Botanical Garden “Jevremovac”, Faculty of Biology, University of Belgrade Studentski trg 16, 11000 Belgrade, Serbia; ekabas@bio.bg.ac.rs (E.K.); sneza@bio.bg.ac.rs (S.V.); majat@bio.bg.ac.rs (M.L.)

**Keywords:** *Astragalus albicaulis*, new finding, Dimitrovgrad, isolated population, chromosome number, steppic grasslands

## Abstract

A yellow-flowered *Astragalus* from section *Dissitiflori* DC. is reported for the first time for the flora of the central Balkan Peninsula, near the city of Dimitrovgrad (E Serbia). This finding significantly expands the known distribution range of yellow-flowered representatives of this group. The taxonomy and nomenclatural status of yellow-flowered taxa of *Astragalus* sect. *Dissitiflori* in Europe remain problematic and partly unresolved due to considerable morphological variation, overlapping morphological and chorological characters, limited molecular and ecological data, and an insufficient number of comparative studies. To clarify the taxonomic position of the newly discovered population, we provided a comparative overview of morphological characters of closely related taxa, determined the chromosome number (2*n* = 4*x* = 32), and provided data on habitat characteristics, ecology and population status. Based on the most recent taxonomic concepts, the population from Dimitrovgrad is referred to as *Astragalus albicaulis*. However, further comprehensive morphological, anatomical, palynological, karyological, and ecological studies are required for its precise taxonomic and nomenclatural delimitation within sect. *Dissitiflori*.

## 1. Introduction

*Astragalus* L. is one of the largest flowering plant genera, comprising c. 2400 to 3000 species classified into more than 240 sections worldwide [1,2,3]. A relatively large number of taxa of the genus *Astragalus* (c. 60) are present in the flora of the Balkan Peninsula, while in Serbia 19 species have been reported up to now [4,5].

One of the most numerous sections in the genus *Astragalus* is sect. *Dissitiflori* DC. with c. 50 species in Europe [3]. The taxonomy and nomenclatural status of different representatives of this group in Europe are still problematic and partly unresolved, due to considerable morphological variation, morphological and chorological overlapping, restricted molecular and ecological research, and an insufficient number of comparative studies. This is clearly evident when comparing historical taxonomic treatments of this group with more recent concepts and results presented by Podlech [6,7], Podlech and Zarre [8], Bartha et al. [3,9], and Kurtto [10]. These problems are also reflected in the yellow-flowered (whitish to yellow) representatives of the sect. *Dissitiflori* present in the Balkan Peninsula and adjacent regions, such as *A. vesicarius* L. s.l., *A. albicaulis* DC., A. *dealbatus* Pall., *A. glaucus* M. Bieb., *A. peterfii* Jáv., *A. pseudoglaucus* Klokov (=*A. vesicarius* subsp. *pseudoglaucus* (Klokov) Ciocârlan), and *A. tarchankuticus* Boriss. Molecular and morphometric studies have further highlighted both close relationships and substantial morphological overlap among several of these taxa [3,9].

The taxonomic complexity of this group is also reflected in its cytological variation. Published chromosome counts range from diploid (2*n* = 16) to octoploid (2*n* = 64) among taxa currently assigned to the *A. vesicarius* group and related yellow-flowered taxa [3,11,12,13,14,15,16,17,18]. Such variation, together with inconsistent taxonomic treatment, emphasizes the need for integrative studies combining morphological and cytological evidence.

**Figure 1:**
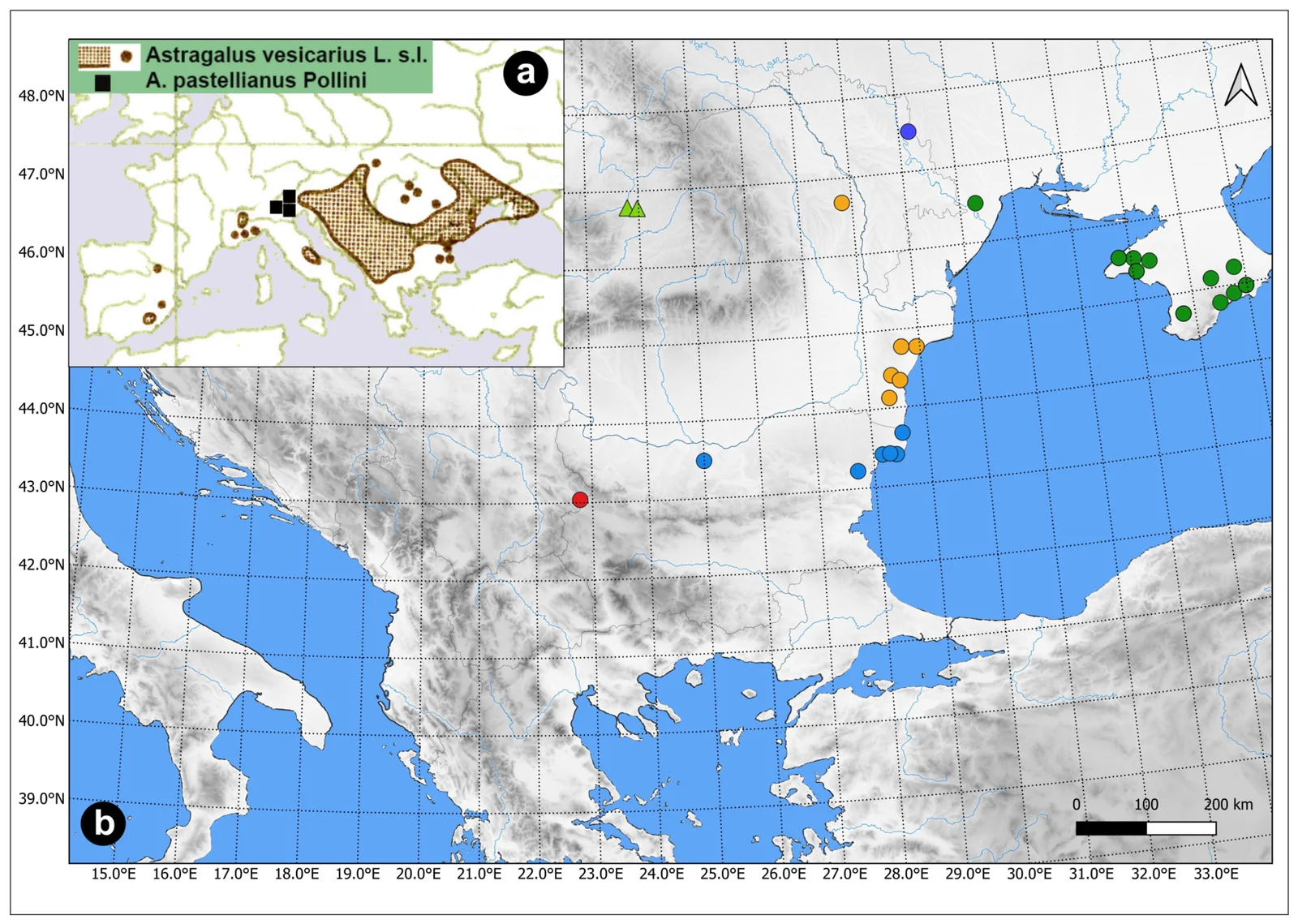
(**a**) *Astragalus vesicarius* s.l.—general distribution (by Meusel et al. [19]—slightly modified). (**b**) Distribution of the first record of *A. albicaulis* in the central Balkan Peninsula and Serbia (red dot), in context of the distribution of its closest and neighboring yellow-flowered relatives (sub. *A. glaucus*—blue dots; *A. glaucus* incl. *A. dealbatus* and *A. tarchancuticus*—green dots; *A. pseudoglaucus*—orange dots; *A. peterfii*—green triangle [8,9,20,21,22,23], modified; total distribution incomplete and unresolved).

In accordance with above-mentioned taxonomic issues, we present here the very first record of a yellow-flowered taxon of *Astragalus* sect. *Dissitiflori*, discovered deep within the Balkan Peninsula (near Dimitrovgrad in E Serbia) (Figure 1), together with data on its morphology, karyology, habitat, ecology and population status.

## 2. Results

### 2.1. Morphological Characteristics and Chromosome Number of the A. albicaulis from Dimitrovgrad (E Serbia)

**Figure 2:**
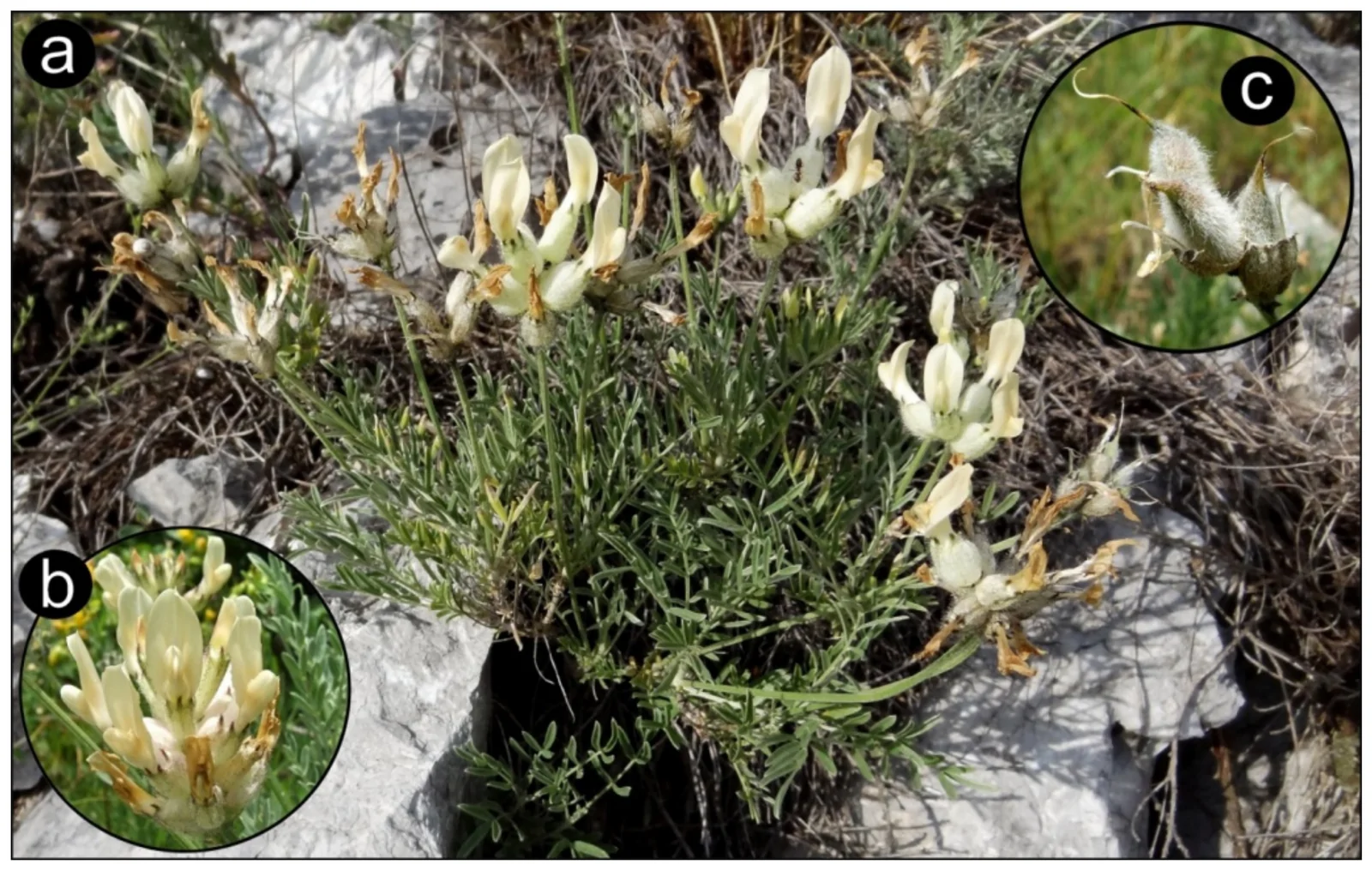
*Astragalus albicaulis* from Dimitrovgrad (E Serbia): (**a**) habitus, (**b**) inflorescence, and (**c**) fruits.

**Figure 3:**
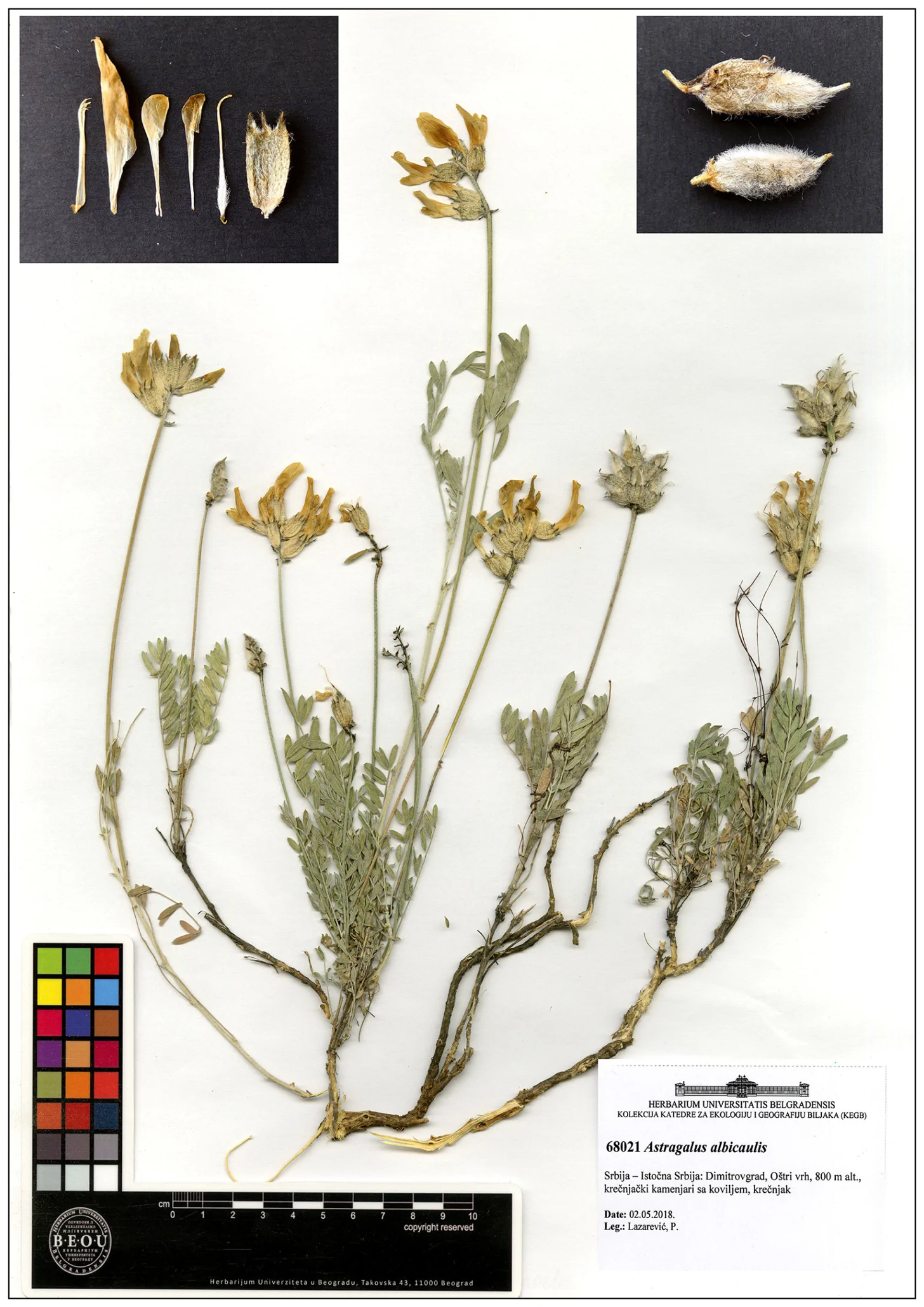
*Astragalus albicaulis* from Dimitrovgrad (E Serbia): Herbarium specimen and details of flower and legume—slightly enlarged.

**Figure 4:**
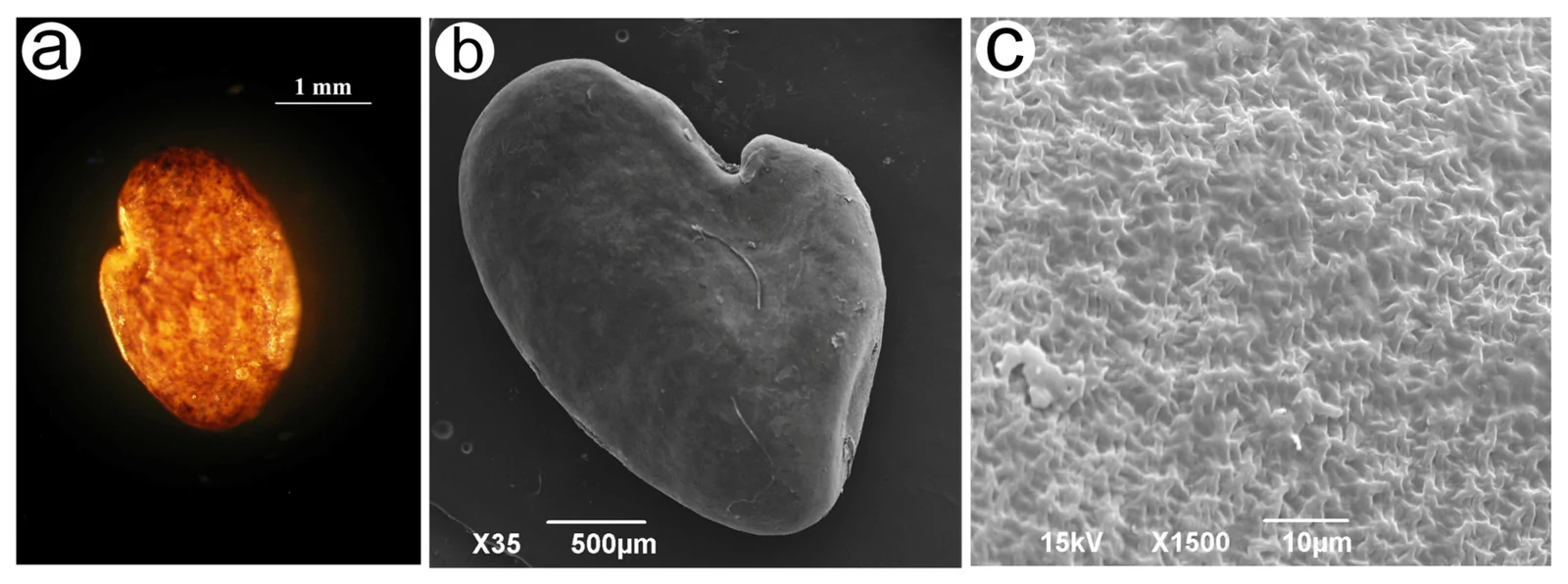
Seeds of *Astragalus albicaulis* from Dimitrovgrad: (**a**) LM photograph; (**b**) SEM microphotograph—whole seed; (**c**) SEM—detail of the surface.

**Figure 5:**
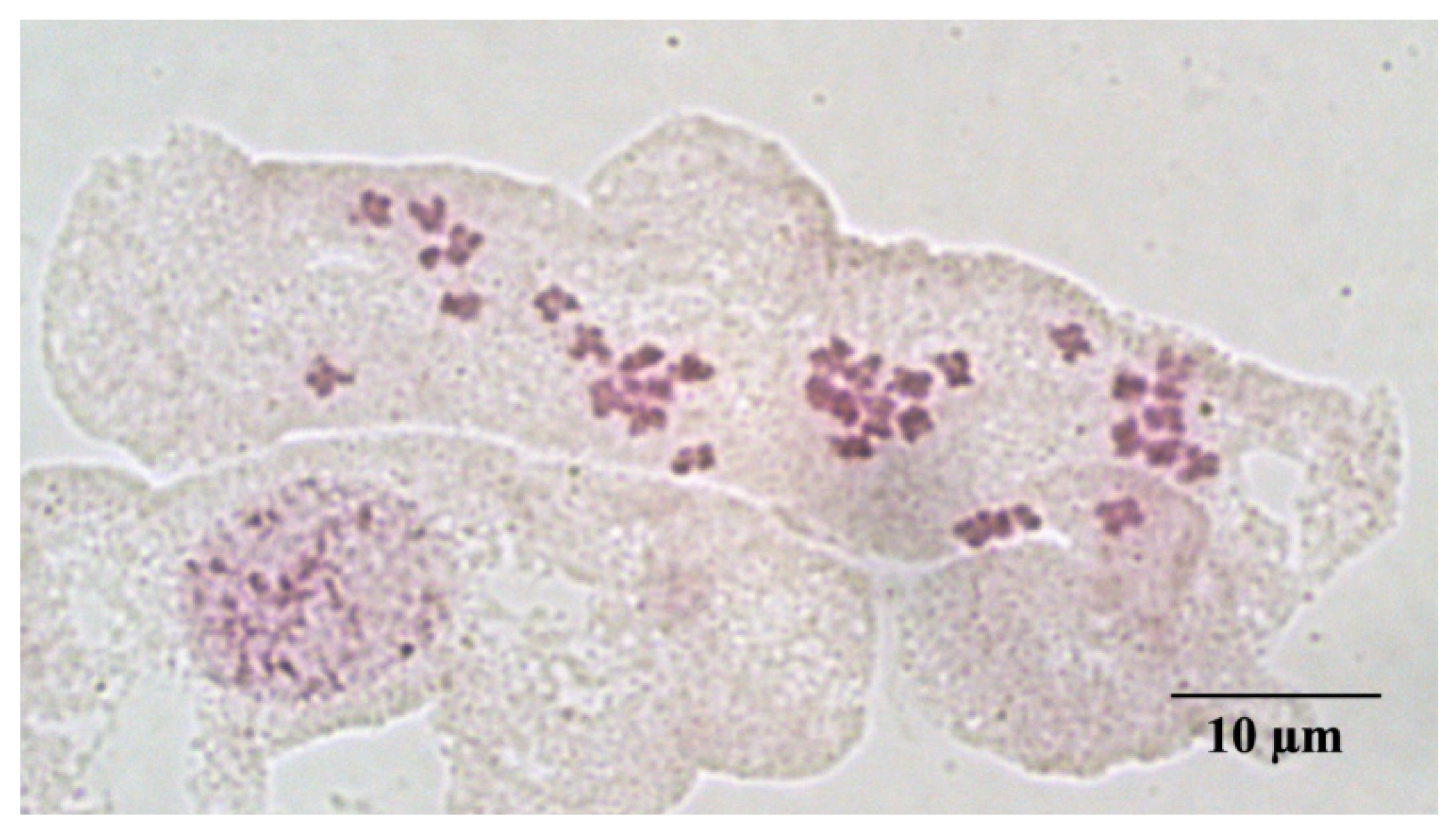
Metaphase chromosome plate *of Astragalus albicaulis* from Dimitrovgrad—2*n* = 4*x* = 32.

Semi-shrub 20–30 (35) cm tall, densely covered with predominantly appressed white hairs giving the plant a silvery-green appearance (Table 1, Figure 2 and Figure 3); rootstock strong with brown bark; leaflets in (4) 5–7 pairs, dimension (6) 10–15 (22) × 2–3 (4) mm, peduncles 10–21 cm long, stipules 3–4 mm long; raceme capitate with 5–11 flowers; calyx tubular to inflated, tube 9–11 mm long with ascending to spreading white hairs reaching up to 1.5 mm and shorter appressed black hairs, calyx teeth 2–3 mm; petals uniform whitish to yellow, standard 21–26 mm long, emarginated and notch base slightly withdrawn back; bracteole 3–4 mm; legumes 12–15 mm long, slightly overgrowing calyx, rostrum 2–3 mm (Table 1, Figure 2 and Figure 3); seeds asymmetrical, reniform or cordiform, 2–3.4 × 1.4–2.4 mm, surface orange-brown to dark-brown, often dark-spotted (Figure 4a,b), seed coat ornamentation rugulate-reticulate (Figure 4c); chromosome number 2*n* = 4*x* = 32 (Table 1, Figure 5).

### 2.2. Taxonomic Key for Most Close Relatives of Astragalus from Dimitrovgrad for Balkans and Adjacent Area, Extracted from Podlech and Zarre [8]

1. Calyx covered with appressed hairs, sometimes with few ascending hairs mixed in (*A. vesicarius*), but then calyx slightly inflated…................................................................ 2

–Calyx covered at least with partly ascending hairs, not only with appressed to subapressed hairs.......................................................................................................................... 4

2. Calyx 9–12 mm, at least partly with spreading, mostly strongly asymmetrically bifurcate to basifixed hairs, tube slightly inflated; standard purple-violet, wings and keel whitish-yellowish [Central & S. Europe]...............................*A. vesicarius* L. subsp. *vesicarius*

–Calyx up to 10 mm, tubular, covered with appressed hairs or more rarely spreading hairs up to 1 mm………………………………………….………………………………………3

3. Petals deeply violet; calyx predominantly covered with mostly appressed black hairs and only few ascending white hairs [NE Italy, Slovenia, Croatia]………..……………………...……...*A. vesicarius* L. subsp. *carniolicus* (A. Kerner) Chater

–Petals yellowish [N. Italy].................*A. vesicarius* subsp. *pastellianus* (Pollini) Arcang.

4. Leaflets linear, c. 10 times longer than wide; leaflets in 5–7 pairs; bracts 2–3 mm, predominantly black hairy; calyx 10–12 mm; petals pale yellowish or whitish yellow, standard blade constricted above the base; legumes 5–24 mm long, beside the strongly asymmetrically bifurcate white hairs 1–1.5 mm also with much shorter black hairs [Romania]...............................................................................................................……*A. peterfii* Jáv.

–Leaflets narrowly elliptic, 3–5 times longer than wide; calyx 10–12 mm; petals whitish-yellow; standard limb oblong-obovate, constricted in lower third: legumes 10–15 mm, with a beak 1–2 mm, covered with ascending to spreading hairs [SE. Europe] .................................................................................................................................*A. albicaulis* DC.

### 2.3. Habitat Description, Ecology and Population Status

*Astragalus albicaulis* from Dimitrovgrad inhabits the south exposed slope of the peak “Oštri vrh”, at 790–820 m a.s.l., represented by dry subcontinental calcareous steppic grasslands with *Stipa eriocaulis* Borbás (Stipetum eriocauli type). The thermophilic and steppic characters of locality “Oštri vrh” and surroundings are well emphasized by the presence of the following species: *S. eriocaulis*, *Saponaria glutinosa* M. Bieb., *Ajuga laxmannii* (Murray) Benth., *Paeonia peregrina* Mill., *Haplophyllum suaveolens* (DC.) G. Don, *Asparagus officinalis* L., *Astragalus onobrychis* L., *Asyneuma anthericoides* (Janka) Bornm., *Chrysopogon gryllus* (L.) Trin., etc. Locality is completely isolated and distant from the nearest yellow-flowered relatives distributed around the Black Sea coast (NE Bulgaria, Romania).

The habitat area covers up to 1 ha and the population is roughly estimated on *c*. 1000 individuals. Since the discovery in 2010, the *A. albicaulis* population from Dimitrovgrad was regularly monitored, and the wider area was searched for new potential localities, but with no new findings. Such a small number of individuals, a very restrictive occupied area, obvious geographic isolation from the closest yellow-flowered relatives, and potential habitat overgrowth and destruction set the requirement for an adequate national conservation, including in situ and ex situ measures. Having all this in mind, the population would qualify for assessment as Critically Endangered CR B1ab(i,ii,iii) + 2ab(i,ii,iii) according to IUCN criteria [24]. Abandonment of traditional uses of grassland (overgrowth) and the potential expansion of the existing military facility located right next to the habitat of species are recognized as major threatening factors.

## 3. Discussion

### 3.1. Taxonomic and Morphological Interpretation of the Dimitrovgrad Population

According to the taxonomic concept established by Podlech [6,7] and Podlech and Zarre [8], *A. albicaulis* is treated in a broad sense, encompassing several previously described taxa, including *A. glaucus*, *A. dealbatus*, and *A. tarchankuticus*. This interpretation unifies earlier, more narrowly defined taxa [25,26,27,28] and considerably expands both the morphological circumscription and the geographic range of *A. albicaulis*. In this broad sense, *A. albicaulis* is reported from Bulgaria, Romania, Moldavia, Ukraine, Russia (European part), and the Caucasus region [8,29]. The morphological characteristics of the yellow-flowered *Astragalus* population discovered near Dimitrovgrad correspond to the descriptions of *A. albicaulis* provided by Podlech [6] and Podlech and Zarre [8].

The morphological evaluation of the *Astragalus albicaulis* population from Dimitrovgrad reveals a definitive taxonomic affinity to *Astragalus albicaulis* DC., despite showing certain variations that merit discussion within the context of intraspecific plasticity. When compared against the comprehensive taxonomic data from Table 1, the population from Dimitrovgrad displays an overwhelming overlap in diagnostic morphological characters with *A. albicaulis*, which firmly distinguishes it from closely related taxa such as *A. peterfii* and *A. vesicarius* sensu lato. Attributes including the characteristic mix of long ascending white and short appressed black hairs on the calyx tube, the distinct emarginate to bilobed shape of the vexillum and the precise calyx teeth lengths of 2–3 mm align perfectly with the established profile of *A. albicaulis*. Furthermore, the qualitative characteristics of the legumes, which feature a specific proportion of ascending white and sometimes black hairs, reinforces this classification and clearly separates the material from Dimitrovgrad from *A. peterfii*, which is characterized by significantly longer (15–24 mm), early-glabrescent legumes and structurally distinct, oblong to obovate vexillum profiles. Similarly, the whitish to yellow corolla color of the Dimitrovgrad plants immediately prevents confusion with *A. vesicarius* reported for Balkan (*A. vesicarius* ssp. *vesicarius* and *A. vesicarius* ssp. *carniolicus*), which is defined by its purplish to violet uniform or bicolor flowers and densely villous calyx indumentum.

Nevertheless, the Dimitrovgrad population is characterized by a suite of localized, divergent features such as the following (Table 1): the specimens display predominantly black-hairy bracts and a marked inflation of the calyx at the end of anthesis, contrasting with the mixed white and black hairs and more strictly tubular to slightly inflated calyx configuration noted in typical *A. albicaulis*. Plants from Dimitrovgrad manifested the lower plant height (20–30 cm) and smaller flower counts per raceme (5–11). Additionally, the presence of spreading white hairs on the calyx, reaching up to 1.5 mm in length, slightly exceeds the limits given in the available keys and descriptions, where calyx hairs are described as ascending and not exceeding 1 mm in length [6,8]. Rather than warranting a separate species status, we interpret these specific shifts as a localized adaptation. This disjunct positioning on the Balkan Peninsula indicates a degree of morphological divergence within *A. albicaulis*.

### 3.2. Chorological Context and Comparison with Neighboring Populations

Within the framework of Podlech’s taxonomic treatment, *Astragalus vesicarius* s.l. comprises three subspecies: subsp. *vesicarius* and subsp. *carniolicus*, both characterized by purplish to violet uniform or bicolor flowers, and subsp. *pastellianus*, with yellowish to yellow flowers [6,8,10].

Based on the provided identification keys and species descriptions [6,8], the studied population from Dimitrovgrad is referred to here as *A. albicaulis*, which differs from representatives of *A. vesicarius* s.l. as follows. From subsp. *vesicarius* and subsp. *carniolicus* it differs by its uniform whitish to yellow petals; from subsp. *carniolicus* and subsp. *pastellianus* additionally by calyx exceeding 10 mm (vs. calyx 8–10 mm). Subsp. *pastellianus*, which shares petal colors with *A. albicaulis*, is further distinguished by its restricted endemic distribution in the western and central Alps, in contrast to the SE European-Caucasus range of *A. albicaulis*.

The Dimitrovgrad population clearly belongs to the group of yellow-flowered representatives of sect. *Dissitiflori*. Geographically, it represents an isolated occurrence, situated far to the west of the nearest known yellow-flowered populations in Bulgaria and Romania. This pronounced geographic isolation, combined with the steppe-like ecological conditions of the locality, suggests that the population from Dimitrovgrad represents a relict occurrence linked to the former, wider distribution of steppe and forest-steppe habitats in the Central Balkans [30,31,32].

At the national and regional level, the interpretation of yellow-flowered *Astragalus* taxa has been inconsistent. In Bulgaria, plants traditionally referred to as *A. glaucus* are mostly recorded from the northeastern Black Sea region [15,22,33,34,35] (Figure 1). These populations are treated as *A. albicaulis* by Podlech [6,7] and Podlech and Zarre [8], whereas other authors consider them to belong to *A. pseudoglaucus* or *A. vesicarius* subsp. *pseudoglaucus* [3,36].

Similar taxonomic uncertainty is evident in Romania, where recent treatments recognize *A. pseudoglaucus* and *A. peterfii*, while rejecting the presence of *A. glaucus* [3,9,37,38] (Figure 1). Furthermore, Euro + Med PlantBase [39] excludes *A. glaucus*, *A. pseudoglaucus* and *A. albicaulis* from Bulgaria and Romania, placing Moldova as the southwestern distribution limit of all three taxa, whereas Plants of the World Online [29] accepts the presence of *A. albicaulis* in Romania and Bulgaria and treats *A. pseudoglaucus* as a synonym of *A. vesicarius* subsp. *vesicarius*, with a range extending from E Central and SE Europe to Ukraine.

Considerable taxonomic, nomenclatural, and chorological inconsistencies are clearly evident for the taxa reported as *A. pseudoglaucus* in Romania. It is important to note that Podlech [7] and Podlech and Zare [8] treated yellow colored petal taxon *A. pseudoglaucus* as a synonym of *A. vesicarius*. Controversially, they did not accept the proposal from Chater [40] in Flora Europaea for *A. pseudoglaucus* “perhaps to be included” in *A. vesicarius* subsp. *pastellianus,* the only yellow-flowered representative of the *A. vesicarius* agg.

Furthermore, molecular studies by Bartha et al. [3] implied a close phylogenetic affinity between *A. pseudoglaucus* and *A. pallescens* M. Bieb., *A. peterfii*, and *A. tarchankuticus*, which Kurtto [10] subsequently designated as the *A. pallescens* group. However, their molecular datasets yielded somewhat conflicting evolutionary scenarios depending on the genetic markers utilized. While plastid DNA sequences preferentially cluster the yellow-flowered representatives, linking *A. pseudoglaucus* with *A. albicaulis* and *A. vesicarius* subsp. *pastellianus,* nuclear ribosomal ITS data reveal a contrasting topology where *A. albicaulis* groups alongside *A. vesicarius*, whereas *A. pseudoglaucus* aligns with *A. tarchankuticus* and *A. peterfii*. Particularly indicative of this complexity is that, contrary to molecular evidence, multivariate morphometric analyses within *Astragalus* sect. *Dissitiflori* [9] failed to establish a clear phenotypic separation between *A. pseudoglaucus* and *A. vesicarius*, establishing a substantial morphological overlap instead. This is further corroborated by Podlech [6] and Podlech and Zarre [8], who noted introgressions between subsp. *vesicarius* and subsp. *carniolicus* in the contact areas of Western Balkans, and probably transitions of *A. vesicarius* to *A. albicaulis* in Romania.

In Serbia, historical records of *A. vesicarius* date back to Pančić [41] on Mt. Suva Planina. He noted that flower color can be bluish to rarely yellowish, but did not explicitly document fully yellow-flowered populations. Later, Diklić [4] reported *A. vesicarius* subsp. *pastellianus* from Mt. Suva Planina, describing only violet flowered plants. In this context, the Dimitrovgrad population of *A. albicaulis* represents the first well-documented occurrence of a yellow-flowered *Astragalus* from sect. *Dissitiflori* in Serbia.

**Figure 6:**
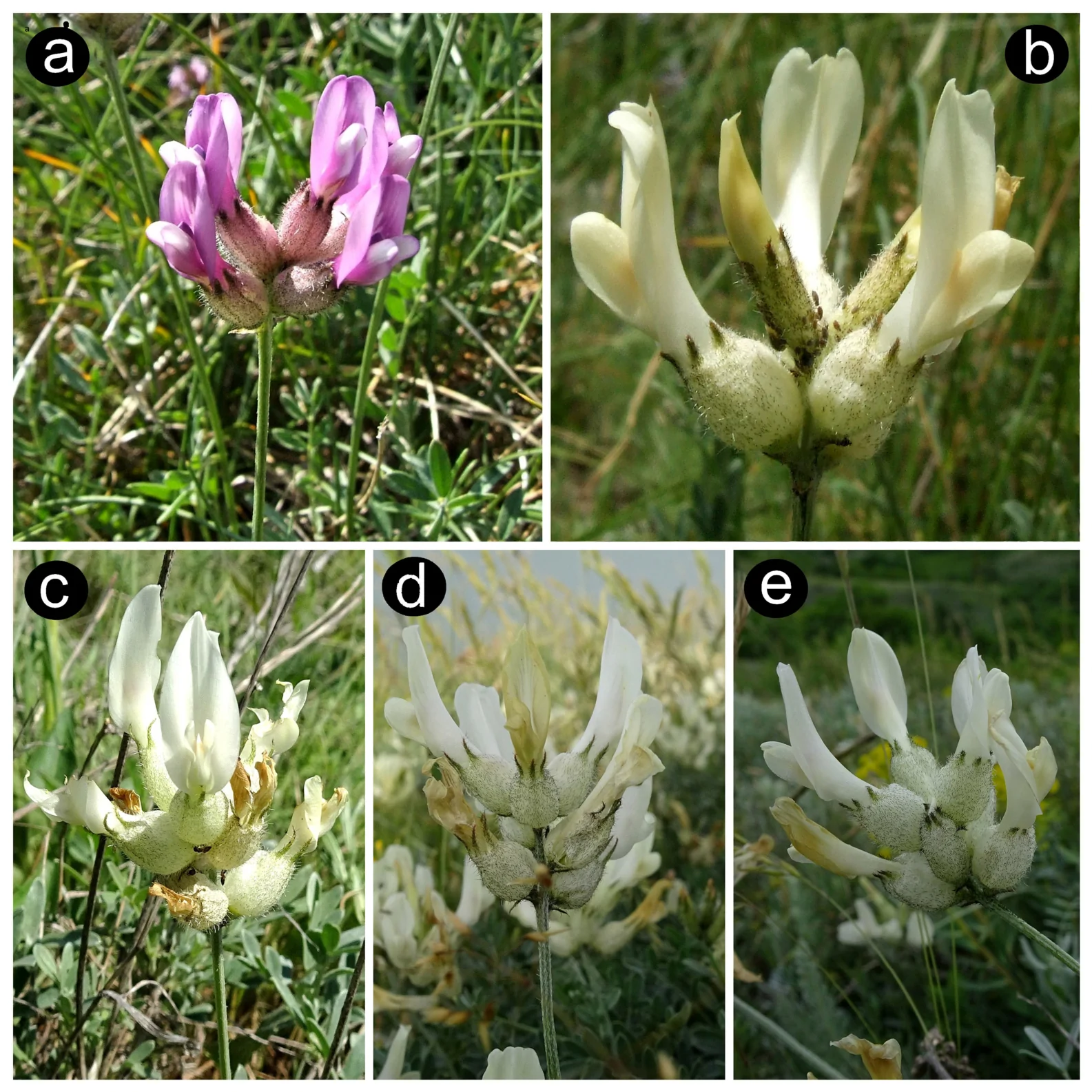
Comparative overview of petal colors of (**a**) *A. vesicarius* subsp. *carniolicus*—Mt. Suva Planina (Serbia); (**b**) *A. albicaulis*—Dimitrovgrad (Serbia); (**c**) *A. pseudoglaucus*—Babadag (Romania); (**d**) *A. pseudoglaucus*—Jurilovca (Romania); and (**e**) *A. glaucus*—Balchik region (Bulgaria).

The petal color is a significant diagnostic character within sect. *Dissitiflori*. The comparative overview presented in Figure 6 illustrates the differences and similarities in flower color between the Dimitrovgrad population and its closest relatives from neighboring regions.

### 3.3. Relictness and Threat Status in Correlation with Neighboring Populations

Pronounced geographic isolation, combined with the steppe-like ecological conditions of the locality, suggests that the population from Dimitrovgrad represents a relict occurrence linked to the former, wider distribution of steppe and forest-steppe habitats in the Central Balkans [32]. In an ecological and biogeographical sense, the habitat where the new record was noticed (as well as the other similar habitats in the Balkan Peninsula and southern parts of Central Europe) has the status of relic extrazonal steppe habitats [30,31].

The newly discovered *A. albicaulis* population on the Balkan Peninsula is qualified as Critically Endangered—CR, while the threat status among neighboring related populations reflects the taxonomic and nomenclatural confusion within this group. In Bulgaria, the conservation status of *A. glaucus* was assessed as EN [22]. In Romania, *A. peterfii* was assessed as CR; neither *A. pseudoglaucus* nor *A. vesicarius* s.l. has been evaluated, while *A. glaucus* was assessed as R [42]. In Moldova, the taxon *A. pastellianus* was proposed for inclusion in the national Red Book under the category EN [43]. In Ukraine, the taxon *A. glaucus* is listed under the category VU [20]. For the Alpine region, the population of *A. vesicarius* subsp. *pastellianus* was included in the French Red List under the category EN [44].

### 3.4. Chromosome Numbers and Their Taxonomic Implications

The long-standing taxonomic confusion within sect. *Dissitiflori* is also reflected in the wide range of reported chromosome numbers for taxa traditionally assigned to the *A. vesicarius* group. Published data include diploid (2*n* = 2*x* = 16), tetraploid (2*n* = 4*x* = 32), and even octoploid (2*n* = 8*x* = 64) cytotypes, often under different species names and without consistent taxonomic concepts (Table 2). For example, Bartha et al. [3] cite 2*n* = 8*x* = 64 for *A. pseudoglaucus* in Romania, referring to *A. glaucus* in Bulgaria and considering them the same taxon.

In this context, the chromosome number determined for the Dimitrovgrad population (2*n* = 4*x* = 32) falls within the range reported for some populations of *A. vesicarius* subsp. *vesicarius* and some other related taxa. This cytological result neither contradicts nor unequivocally confirms its assignment to *A. albicaulis* sensu Podlech, but it provides an important additional data point for future comparative studies. Together with morphological and chorological evidence, the karyological data underline the need for integrative taxonomic approaches combining morphology, cytogenetics, ecology, and molecular data to resolve species boundaries within sect. *Dissitiflori*.

All of the above implies taxonomic, nomenclatural, and chorological inconsistencies in the interpretation within this group. For more appropriate delineation of *Astragalus* sect. *Dissitiflori*, larger comprehensive morphological, anatomical, palynological, karyological and ecological studies are needed.

## 4. Materials and Methods

The newly discovered and hitherto the only known locality of the *A. albicaulis* in Serbia was for the first time recorded on 12 May 2010 near Dimitrovgrad (C Balkan Peninsula) (Figure 1). The voucher specimen (voucher number 17480) is deposited in the Herbarium of the University of Belgrade—BEOU (acronym according to Thiers [45]). The main morphological characteristics of 20 individuals from this population were measured and assessed. The measured traits included vegetative and reproductive characters (plant height, leaflet number and dimensions, peduncle and stipule length, number of flowers per raceme, calyx length, standard length, legume length and rostrum length), as well as the indumentum characteristics (hair type, orientation and length). These traits were selected based on their diagnostic value in the identification keys used for comparison with related taxa. Additionally, the size and general morphology of 50 seeds of *A. albicaulis* were investigated using a LEICA MZ7.5 stereo-binocular microscope (Leica Microsystems, Wetzlar, Germany). Seeds’ micromorphological characteristics were studied using a scanning electron microscope (JEOL JSM-6390LV, JEOL Ltd., Tokyo, Japan) on samples coated with gold for 100 s.

Mitotic chromosome number was determined from at least 10 individuals using apical root meristems obtained from germinated seeds. Roots were pretreated with 0.002M 8-hydroxyquinoline for 3 h at 4 °C. Fixation was done in a cold 3:1 mixture of absolute ethanol and glacial acetic acid for 48 h, hydrolysis in 1N HCl for 12 min at 60 °C, and staining in Schiff reagent for 3 h [46]. Final squash was completed using acetic carmine. Chromosome plates were observed on a Leica DMLS light microscope (Leica Microsystems, Wetzlar, Germany) and photographed using a Leica DCF 295 camera.

## 5. Conclusions

This study documents the first record of a yellow-flowered representative of *Astragalus* sect. *Dissitiflori* for the Central Balkan Peninsula, based on an isolated population discovered near Dimitrovgrad (E Serbia). On the basis of morphological characters and their chorological context, this population is considered *Astragalus albicaulis* sensu Podlech.

The morphological evaluation of the *Astragalus* population from Dimitrovgrad reveals a definitive taxonomic affinity to *Astragalus albicaulis* DC., despite showcasing unique phenotypic variations that were discussed within Section 2. When compared against the comprehensive taxonomic treatment by Podlech and Zare [8], the population from Dimitrovgrad displays an overwhelming overlap in diagnostic traits, which firmly distinguishes it from closely related taxa such as *A. peterfii* and *A. vesicarius* sensu lato (ssp. *vesicarius* and ssp. *carniolicus*).

At the same time, some morphological deviations and the broader taxonomic instability within sect. *Dissitiflori* indicate that species boundaries among yellow-flowered taxa of the *A. vesicarius* group remain insufficiently resolved. The Dimitrovgrad population therefore represents an important case study highlighting the need for integrative taxonomic approaches combining morphological, cytogenetic, ecological, and molecular data.

In addition to its taxonomic relevance, the population is of considerable conservation importance, as a geographically isolated and relict occurrence restricted to a very small area. Given its limited population size, habitat specificity, and identified threats, appropriate conservation measures at the national level are therefore strongly recommended.

## Figures and Tables

**Table 1:** Comparative table of the differential morphological characters of *A. albicaulis* from Dimitrovgrad and most closely related *Astragalus* taxa retrieved from Podlech and Zarre [8].

Morphological Character	*A. albicaulis* *(Dimitrovgrad)*	*A. albicaulis* DC.	*A. peterfii* Jáv.	*A. vesicarius* L. s.l.
**Plant habitus**	Semi-shrub	Subshrub	Subshrub	Subshrub
**Plant height (cm)**	20–30 (–35)	20–40 (–50)	30–40	35
**Stipule length (mm)**	3–4	3–6	(3–) 4–6	2–3
**Stipule indumentum**	Densely white hairy, sometimes with some black hairs, predominantly at the base	Loosely to rather densely white hairy, sometimes mixed with black hairs	Rather densely white hairy, sometimes mixed with black hairs	Densely hairy
**Number of leaflet pairs**	(4) 5–7	3–5 (6) (longest leaflets up to 25–30 mm)	5–7	(3–) 5–10
**Leaf length and width (mm)**	(6) 10–15 (–20) × 2–3 (–4)	6–25 × 1–6 (–10)	10–30 × 0.5–3.5	4–12 × 1.1–3
**Leaf indumentum**	Densely covered with appressed silver hairs on both sides	Densely silvery or more rarely sparsely to loosely appressed hairy on both sides	Densely hairy like the stem	Mostly rather densely, rarely only loosely hairy
**Peduncle length (cm)**	10–21	10–25	12–25	3–19
**Peduncle indumentum**	White hairy, toward the raceme also with black hairs	White hairy, toward the raceme also with black hairs	Loosely to more rarely rather densely white hairy, sometimes below the raceme with few black hairs mixed in	White hairs, just below the raceme also with few black hairs
**Number of flowers per inflorescence**	5–11	7–18	10–30	3–20
**Inflorescence length in fruit (cm)**	2.5–5	Up to 9	5–12	Scarcely elongated at fruiting time
**Bract length (mm)**	3–4	3–5	2–3	2.5–5
**Bract indumentum**	Predominantly black hairy	White and black hairs	With asymmetrically bifurcate, predominantly black hairs	Predominantly black hairy
**Calyx length (mm)**	9–11	10–12	10–13	8–12
**Calyx teeth length (mm)**	2–3	2–4	2–4	2–4
**Calyx indumentum**	Ascending white hairs up to 1.5 mm and much shorter appressed, medifixed black hairs, teeth mostly with black hairs	Ascending, white hairs up to 1 mm and much shorter appressed black hairs, sometimes with five blackish lines ending in the teeth	Loosely to rather densely covered with bifurcate, subappressed to ascending white hairs 0.6–1 mm and with few to many subappressed black hairs	Densely villous with spreading white and also black hairs up to 2 mm, sometimes with few, short, nearly appressed black hairs, or predominantly with white and also ± appressed white hairs or nearly exclusively with short black hairs
**Calyx inflation at fruiting**	Inflated	Tubular or slightly inflated	Tubular, slightly obliquely gibbous at the base, slightly obliquely cut at the mouth	Somewhat swollen, more rarely distinctly to elliptically inflated
**Petal color**	Whitish to yellow	Whitish to yellow	Pale yellowish or whitish-yellow	Purplish to violet or (particularly in the eastern part of its area) wings and keel often whitish
**Vexillum length (mm)**	(21) 22–25 (–26)	(18) 20–27	18–24	17–23
**Vexillum shape**	Emarginate to bilobed	Emarginate to bilobed	Oblong to obovate, rounded to truncate, slightly constricted above the base	Obovate to slightly panduriform, emarginate, passing into the cuneate claw
**Wing length (mm)**	17–19	17–20	18–20	15.5–20 (–21.5)
**Keel length (mm)**	16–17	15–17	15–17	13–16 (–18)
**Legume length (mm)**	10–12	10–15	15–24	8–15
**Legume rostrum length (mm)**	2–3	1–2	2–4	2–3
**Legume indumentum**	Predominantly with ascending and spreading white hairs, and also with some black hairs	White hairs	Rather densely covered with ascending to spreading white hairs 1–1.5 mm and with much shorter, subappressed to ascending black hairs, soon glabrescent and finally often nearly glabrous	Covered sparsely with short basifixed to medifixed appressed black hairs and densely with basifixed, villous white hairs up to 1.2 mm, sometimes the black hairs missing

**Table 2:** Chromosome numbers (2*n*) reported for the selected taxa from sect. *Dissitiflori.*

Taxon *	2*n*	Ploidy Level (*x*)	Country	Reference
*Astragalus albidus* Waldst. & Kit.	16	2	Slovakia	[16]
*Astragalus glaucus* M.Bieb.	64	8	Bulgaria	[14]
*Astragalus peterfii* Jáv.	64	8	Romania	[17]
*Astragalus vesicarius* L.	16	2	Bulgaria	[13]
	32	4	Bulgaria	[14]
*Astragalus vesicarius* L. subsp. *albidus* (Waldst. & Kit.) Br.-Bl.	16	2	Slovakia	[11]
*Astragalus vesicarius* L. subsp. *carniolicus* (A. Kern.) Chater	16	2	Croatia	[18]
*Astragalus vesicarius* L. subsp. *pastellianus* auct.	32	4	Bulgaria	[15]
*Astragalus vesicarius* L. subsp. *vesicarius*	16	2	Spain	[12]
	32	4	Bulgaria	[15]

* Original species names used in the articles where they were published.

## Data Availability

The original contributions presented in this study are included in the article. Further inquiries can be directed to the corresponding author.

## References

[1] Lock J.M., Schrire B.D., Lewis G., Schrire B., Mackinder B., Lock M. (2005). Galegeae. Legumes of the World.

[2] Hardion L., Baumel A., Dumas P.J., Duong N., Affre L., Tatoni T. (2010). Phylogenetic relationships and infrageneric classification of *Astragalus tragacantha* (Fabaceae), inferred from nuclear ribosomal DNA Internal transcribed data spacers data (*nrDNA ITS*). Ecol. Mediterr..

[3] Bartha L., Dragoș N., Molnár A.V., Sramkó G. (2013). Molecular evidence for reticulate speciation in *Astragalus* (Fabaceae) as revealed by a case study from sect. Dissitiflori. Botany.

[4] Diklić N., Josifović M. (1972). *Astragalus* L. Flora SR Srbije 4.

[5] Ranđelović V., Zlatković B., Jušković M. *Astragalus wilmottianus* Stoj., a new species for the flora of Serbia. Proceedings of the 7th Symposium on Flora of Southeastern Serbia.

[6] Podlech D. (2008). The genus *Astragalus* (Fabaceae) in Europe. Feddes Repert..

[7] Podlech D. (2011). Thesaurus Astragalorum I.

[8] Podlech D., Zarre S. (2013). A Taxonomic Revision of the Genus Astragalus (Leguminosae).

[9] Bartha L., Tóth J.P., Molnár A.V., Dragoș N., Sramkó G. (2012). Taxonomic implications of multivariate morphometrics in selected taxa of the genus *Astragalus* sect. *Dissitiflori* (Fabaceae). Contrib. Bot..

[10] Kurtto A. (2017). Leguminosae (Fabaceae): Astragalus to Erophaca. Atlas Florae Europaeae 19 (Draft).

[11] Murín A., Májovský J.M. (1976). IOPB chromosome number reports LIII. Taxon.

[12] Pretel Martínez A. (1974). IOPB chromosome number reports XLVI. Taxon.

[13] Kožuharov S.I., Petrova A.V., Markova T. (1973). IOPB chromosome number reports XL. Taxon.

[14] Kuzmanov B. (1993). Chromosome numbers of Bulgarian angiosperms: An introduction to a chromosome atlas of the Bulgarian flora. Flora Medit..

[15] Pavlova D., Kožuharov S. (1993). Chromosome numbers of Bulgarian angiosperms. Fitologija.

[16] Májovský J., Uhríková A., Javorčíková D., Mičieta K., Králik E., Dúbravcová Z., Feráková V., Murín A., Černušáková D., Hindáková M. (2000). First supplement to the karyotaxonomic survey of the flora of Slovakia. Acta Fac. Rerum Nat. Univ. Comen. Bot. Suppl..

[17] Lungeanu I. (1975). IOPB chromosome number reports XLIX. Taxon.

[18] Siljak-Yakovlev S., Pustahija F., Šolić E.M., Bogunić F., Muratović E., Bašić N., Catrice O., Brown S.C. (2010). Towards a genome size and chromosome number database of Balkan flora: C-Values in 343 taxa with novel values for 242. Adv. Sci. Lett..

[19] Meusel H., Jäger E., Weinert E. (1965). Comparative Chorology of the Central European Flora. Volume 1, Maps.

[20] Kagalo O.O., Didukh Y.P. (2009). Glaucous Milk-vetch *Astragalus glaucus* M.Bieb. (*A. dealbatus* Pall., *A. tarchankuticus* Boriss.). Red Data Book of Ukraine. Plant World.

[21] Izverscaia T. (2012). Notes on some genera *Astragalus* L. (Fabaceae) species in dniester-prit river region. Conservation of plant diversity International Scientific Symposium.

[22] Stoyanov S., Peev D. (2015). *Astragalus* *glaucus*. Red Data Book of the Republic of Bulgaria, Volume 1: Plants and Fungi.

[23] GBIF Occurrence Download.

[24] IUCN (2012). IUCN Red List Categories and Criteria: Version 3.1.

[25] De Candolle A.P. (1802). Astragalogia Nempe Astragali, Biserrulae et Oxytropidis, nec non Phacae, Colutae et Lessertiae, Historiis Iconibus Illustrata.

[26] Bieberstein M.F.A. (1808). Flora Tauro-Caucasica Exhibens Stirpes Phaenogamas, in Chersoneso Taurica et Regionibus Caucasicis Sponte Crescentes.

[27] Bieberstein M.F.A. (1819). Flora Taurico-Caucasica, Supplementum Continens Plantas Phanerogamas.

[28] Borissova A. (1951). *Astragali* et *Tragacantae* Tauriae. Bot. Mater. Gerb. Bot. Inst. Komar. Akad. Nauk SSSR.

[29] POWO. Plants of the World Online.

[30] Mucina L., Bültmann H., Dierßen K., Theurillat J.-P., Raus T., Čarni A., Šumberová K., Willner W., Dengler J., García R.G. (2016). Vegetation of Europe: Hierarchical floristic classification system of vascular plant, bryophyte, lichen, and algal communities. Appl. Veg. Sci..

[31] Kabaš E., Vukojičić S., Aćić S., Lakušić D. (2022). Phytosociology of Stipa-dominated steppe-like vegetation on the ultramafics of the Central Balkans. Bot. Serb..

[32] Chytrý K., Willner W., Chytrý M., Divíšek J., Dullinger S. (2022). Central European forest–steppe: An ecosystem shaped by climate, topography and disturbances. J. Biogeogr..

[33] Vălev S., Jordanov D. (1976). *Astragalus*. Flora Republicae Popularis Bulgaricae 6.

[34] Kožuharov S., Kožuharov S. (1992). *Astragalus* L. Opredelitel na Visshite Rastenija v Bulgaria.

[35] Tzonev R., Roussakova V., Dimitrov M. (2006). The Western-Pontic steppe vegetation in Bulgaria. Hacquetia.

[36] Badarau S., Dezsi S., Comes O. (2000). Cercetări biogeografice asupra speciilor stepice-silvostepice de *Astragalus* L. din Depresiunea Transilvaniei (I). Stud. Univ. Babeș-Bolyai Geogr..

[37] Ciocârlan V., Sârbu I. (2001). Taxonomy, variability and distribution of selected species of *Astragalus* L. in Romania. Bull. Bot. Gard. Iași.

[38] Ciocârlan V. (2009). Flora Ilustrată a României.

[39] Euro+Med Euro+Med PlantBase—The Information Resource for Euro-Mediterranean Plant Diversity. http://www.europlusmed.org.

[40] Chater A.O., Tutin T.G., Heywood V.H., Burges N.A., Valentine D.H., Walters S.M., Webb D.A. (1968). *Astragalus* L. Flora Europaea, Volume 2: Rosaceae to Umbelliferae.

[41] Pančić J. (1884). Additamenta ad Floram Principatus Serbiae.

[42] Hurdu B.-I., Coste A., Halmagyi A., Szatmari P.-M., Farkas A., Pușcaș M., Dan Turtureanu P., Roșca-Casian O., Tănase C., Oprea A. (2022). Ex Situ Conservation of Plant Diversity in Romania: A Synthesis of Threatened and Endemic Taxa. J. Nat. Conserv..

[43] Izverscaia T., Ghendov V., Sirodoev G. (2022). Endangered Spontaneous Fabaceae Species Proposed for Inclusion in the Red Book of the Republic of Moldova. J. Bot..

[44] Franck J. Listes Rouges Françaises. Flore Alpes. https://www.florealpes.com/choix_uicn.php.

[45] Thiers B. Index Herbariorum. New York Botanical Garden. https://sweetgum.nybg.org/.

[46] Feulgen R., Rossenbeck H. (1924). Mikroskopisch-chemischer Nachweis einer Nukleinsäure vom Typus der Thymonukleinsäure. Hoppe-Seyler’s Z. Physiol. Chem..

